# Grip Stabilization through Independent Finger Tactile Feedback Control

**DOI:** 10.3390/s20061748

**Published:** 2020-03-21

**Authors:** Filipe Veiga, Benoni Edin, Jan Peters

**Affiliations:** 1 Computer Science and Artificial Intelligence Laboratory (CSAIL), Massachusetts Institute of Technology, Cambridge, MA 02139, USA; fveiga@mit.edu; 2 Department of Integrative Medical Biology, Umeå University, 901 87 Umeå, Sweden; benoni.edin@umu.se; 3 Intelligent Autonomous Systems Group, Technische Universität Darmstadt, 64289 Darmstadt, Germany; 4 Max-Planck-Institut für Intelligente Systeme, 72076 Tübingen, Germany

**Keywords:** in-hand manipulation, modular control, reactive control, tactile feedback, independent finger control, slip prediction

## Abstract

Grip force control during robotic in-hand manipulation is usually modeled as a monolithic task, where complex controllers consider the placement of all fingers and the contact states between each finger and the gripped object in order to compute the necessary forces to be applied by each finger. Such approaches normally rely on object and contact models and do not generalize well to novel manipulation tasks. Here, we propose a modular grip stabilization method based on a proposition that explains how humans achieve grasp stability. In this biomimetic approach, independent tactile grip stabilization controllers ensure that slip does not occur locally at the engaged robot fingers. Local slip is predicted from the tactile signals of each fingertip sensor i.e., BioTac and BioTac SP by Syntouch. We show that stable grasps emerge without any form of central communication when such independent controllers are engaged in the control of multi-digit robotic hands. The resulting grasps are resistant to external perturbations while ensuring stable grips on a wide variety of objects.

## 1. Introduction

Robotic grasping and in-hand manipulation are traditionally viewed as monolithic planning and control problems. As such, control policies determine the approach strategy and finger placement (contact forces and contact locations) for the entire hand, while considering finger trajectories, force and contact profiles throughout the entire manipulation task [1]. This monolithic formalization requires accurate kinematic, dynamic and contact modeling for the hand, object and contacts between the two along with precise sensing of hand and object position as well as interaction forces. To relax these requirements, several approaches for in-hand manipulation reduce the problem complexity by considering only pinch grasps and manipulating objects by using externally applied forces [2], exploring gravity and arm acceleration effects in conjunction with a passive gripper that ensures that a constant amount of grip force is applied [3] or by exploring gravity while making assumptions about the contact between the gripper and the object [4]. With even stronger assumptions about the contacts between the fingers and the object, trajectory optimization approaches can be used to manipulate objects with more dexterous hands [5]. In practice, in order to find general solutions for more complex tasks, control eventually becomes largely data-driven as accurate models are rarely available and due to the uncertainty associated with addressing all the aforementioned problems through a single controller [6].

Data-driven approaches do not come for free. They either require large training data sets [7,8,9], restrict the tasks to sufficiently similar scenarios [6,10], or rely on low-dimensional representations such as synergies [11] and motion primitives [12] that encode the considered manipulation task. Recent approaches rely on even larger amounts of data to learn tasks in simulation environments where the physical parameters are sampled randomly at the begging of each trial such that the learned policies are transferable to the corresponding real platforms [13,14]. Even with the increase of available data, learned polices still inherently couple the employed degrees of freedom, resulting in solutions that are task- and platform-specific. Furthermore, incorporating tactile feedback from all fingers into a control policy quickly becomes intractable given the dimensionality of the feedback signals. In short, low-level control policies that both deal with uncertainty (e.g., in contact locations and forces) and generalize well beyond a limited set of cases, need to be both data-driven and modular.

Ensuring grip stability is central to both stabilizing an object in the hand and moving an object between stable grip configurations. Classical robotics approaches often rely on measures such as form- or force-closure for assessing grip stability—but with imperfect models and contact/force sensing, using such measures is very challenging. As a result, many researchers have proposed alternative grasp stability measures [15,16,17,18,19] and developed accompanying control strategies. Learning-based approaches for grasping are also abundant with some relying on large amounts of robot trials [20] or synthetic data [21] while others combine learning with analytic grasp metrics [22] or use lower-dimensional sub-spaces to find appropriate hand grasping postures [23]. For a more extensive overview of the grasping and manipulation fields we refer the reader to [24,25].

Human grasping and manipulation appears to be largely data-driven [26] despite relying on feedback signals of huge dimensionality and relatively low control precision when compared to robots. As deduced from several behavioral studies [27,28,29,30], human grasp control strategies seem to be modular and based on local sensing and actuation, rendering the control of the fingers largely independent from each other, i.e., Independent Finger Control [27]. Specific grasps and force distributions appear to emerge from tactile feedback as the fingers interact with objects. Clearly, such an approach would be desirable for robotic grasping and manipulation.

In [31], it is suggested that humans can explore objects with a varying number of digits and that the exploratory procedures used are quickly adapted once the number of available digits changes. On the robotics side, early studies suggested that using tactile feedback on one active finger while having the other fingers remain passive, allows the robot to quickly adapt its grip force [32]. Nonetheless, it is also suggested that when increasing the number of active fingers, the complexity of the controllers would have to increase to be able to manage the force interactions between the fingers. Inspired by progression from one finger over two fingers to the whole hand proposed by [31], by early studies of grasp stability using tactile feedback [32] and by the independent control hypothesis in human grip control by [27,28], we have developed independent control policies based on tactile feedback for each finger that in conjunction generalize grip force regulation from one finger to five fingers.

**Figure 1:**
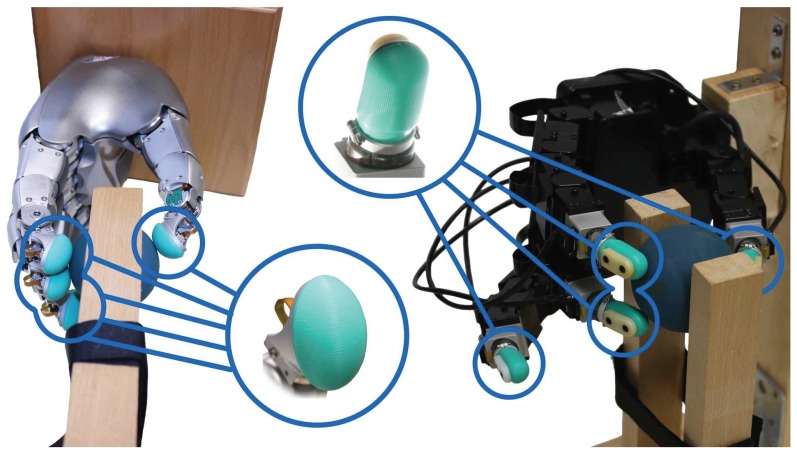
The proposed independent finger grip stabilization controller was successfully evaluated on the four-finger Allegro Hand (right) and on the five-finger Wessling Hand (left). The fingertips of both hands are equipped with Syntouch’s BioTac or BioTac SP sensors, respectively.

To achieve this, we equipped the robotic fingertips of two hands with multimodal fingertip sensors (BioTac and BioTac SP for the four-finger Allegro and five-finger Wessling Hand, respectively; Figure 1), each with a learned predictive model of future slips based on the tactile feedback acquired during finger-object interactions. The local control laws in each finger counteract future slips, ideally preventing them.

The resulting distributed control approach ensures that objects remain withing the grasp. Since the fingers are controlled independently, grasps can be maintained between a finger and other objects (such as a table or a wall), between several robotic fingers (as in in-hand object stabilization or gripping) or between a robotic and a human finger (human-robot joint stabilization). In addition, this approach reproduces findings in human motor control where the absolute amount of force applied by single digits will always settle just above the minimal amount of forces to prevent slip in static settings [26,27]. In more dynamic scenarios, this approach can also facilitate in-hand manipulation by decoupling grip force management from the generation of manipulation trajectories. Manipulation trajectories can also be simplified by considering them as a set of perturbations imposed by one or more fingers while the remaining fingers keep the object stable. While for the static cases, the necessary coordination between independent finger controllers occurs implicitly through the tactile signals observed by each finger, in the dynamic cases some form of explicit coordination may be required. For the latter, the modular nature of the approach is expected to enable higher-level planning systems to operate with less object knowledge while requiring simpler models for control than analytical approaches. This relaxation of model requirements potentially allows for generalization across multiple tasks, across a variety of objects and across different robotic platforms.

## 2. Modular Tactile Sensing-Based Grip Stabilization

As foundation for our modular grip stabilization approach, we start by introducing our previous work on tactile-based slip prediction, validated in the context of single-finger tactile control for stabilizing objects pinned against other objects. Subsequently, we describe how we use the slip prediction information in the multi-finger setting, i.e., fingers of one robot or those of several agents, while also considering potential dynamic scenarios where we wish to reposition the object in-hand.

### 2.1. Slip Prediction

Formulating slip prediction as a classification problem [33], a solution is achieved by training a classifier f(·) that predicts the slip state at time $t+\tau_{f}$, with $\tau_{f}$ representing the prediction horizon. Here, we use $\tau_{f}=10$, corresponding to a prediction of the occurrence of slip 10 time steps or 100 ms prior to its onset. To achieve these predictions, features ϕ(·) of the raw sensor signals $x_{t}\in R^{N}$ were extracted for a time window $T=(t-\tau_{H}):t$, where $\tau_{H}$ is the tactile history and *N* is either 44 or 49 depending on the BioTac version. The feature vector as the form $\left[x_{t},\Delta x\right]$ where $\Delta x=x_{t}-x_{t-1}$. This form of the feature vector considers only the immediate history, corresponding to a value of $\tau_{H}=1$. The slip predictor, i.e., $f(\phi \left(x_{t}\right)$, was trained to correctly label the slip state, *c*, at time $t+\tau_{f}$

(1) $$c_{t+\tau_{f}}=f\left(\phi \left(x_{(t-\tau_{H}):t}\right)\right)$$

as one of the classes in the set $c_{t+\tau_{f}}\in \left\{\mathrm{slip},\mathrm{contact},\neg \mathrm{contact}\right\}$. We used random forests [34] for learning the classifier, achieving good classification rates with a moderately sized data set. Three fingers were made to slide along the surface of a fixed object, while maintaining a specified value of fingertip pressure. Four different objects and nine values of pressure were used, with three repetitions for each object-pressure combination. The data from all three fingers was then merged onto a single data set that was used for each classifier that was trained. A more detailed description of the data acquisition procedure is provided in Section 3.3. For an in-depth study of how the feature function affects the detection and prediction of slip, how each of the individual tactile signals contributes to the detection and prediction accuracies and how such forms of feedback influence the effectiveness of a naive stabilization controller, the reader is referred to our previous work [33].

By learning how to predict slip from the tactile information provided by the BioTac sensors we are able to project valuable information from a 44 or 49-dimensional space onto a one-dimensional discrete variable. As shown in our previous work [33], this form of information generalizes well across objects. These generalization capabilities coupled with the low dimensionality of the classifier outputs will greatly benefit the design of the control approach described in the following section.

### 2.2. Grip Force Control

Human ability to perceptually discriminate forces applied by their fingertips is limited (Weber fractions typically 5–10%, [35]). Accordingly, tactile information types other than those directly related to fingertip force or pressure seem to be employed in human force adjustment strategies during object grasping. As slipping is directly connected to the stability of the interaction with the environment, it is considered crucial for human manipulation [26]. Considering the previously introduced slip prediction approach, grip force management is accomplished through a control law that converts the predicted slip state, *c*, at time $t+\tau_{f}$ into adjustments in the applied normal force. Most robotic hands are controlled in joint or end-effector positions rather than applied forces. To make the controller applicable across a range of robotic hands, our controller therefore adjusted the desired task space velocities, ${\dot{s}}_{t}$, rather than controlling force explicitly. Hence, whenever slip was predicted, we increased the normal force, $F_{N}$, alternatively slowly decreasing the force while keeping the object stable, in line with what has empirically been found during human grasping. This behavior was achieved by using a leaky integrator

(2) $$y_{t}=\alpha y_{t-1}+\left(1-\alpha \right)L$$

to control the task space velocity in the contact normal direction, i.e.,

(3) $${\dot{s}}_{t}=N_{t}y_{t}.$$

Here, α is the leakage at each time step, $y_{t}$ and $y_{t-1}$ are respectively the current and previous states of the integrator, ${\dot{s}}_{t}$ is the task space velocity and $N_{t}$ is a unit vector pointing in the contact normal direction that is estimated every time step from the tactile signals using an heuristic introduced in [36]. The integrator input signal *L* changes with the predicted contact state $c_{t+\tau_{f}}$, increasing the accumulated response when slip is predicted and allowing the integrator to leak if contact is predicted, i.e.,

(4) $$L=\left\{\begin{array}{cc} 1 & \mathrm{if}\;c_{t+\tau_{f}}=\mathrm{slip},\\ 0 & \mathrm{otherwise} \end{array}\right.$$

This integrator thus operates as a smoothing filter which is important given the discrete nature of the slip predictor outputs. In multi-fingered scenarios, any oscillations in the controller response would propagate to other fingers engaged in the grasp and cause instability. While still allowing the fingers to react to all oscillations, the integrators manage the intensity of the response, slightly changing the applied force for instantaneous perturbations or greatly increasing the applied force for more persistent perturbations.

Finally, a minimum integrator response $y_{min}$ is required to avoid oscillations around low integrator responses values where slip is imminent. However, instead of specifying $y_{min}$, each finger estimates its minimum response by observing the first slip transient following a first stable period. The minimum response is then defined as a 10% increase of the response $y_{t}$ where the first transition from contact to slip occurs

(5) $$y_{min}=1.1y_{t}\;\mathrm{if}\;\Delta c=\mathrm{contact}\to \mathrm{slip}.$$

This minimum response implicitly defines the minimum fingertip normal force necessary to prevent slips and makes the controller responsive to the prevailing friction at its digit-object interface.

With this control formalization, each finger is able to regulate its own applied force without requiring any prior information about the object, any reference force value or any explicit information about the force applied by other fingers. In addition, the controllers are able to automatically find the minimal amount of force that the finger has to apply in order to avoid the occurrence of slip by iteratively adjusting the minimum integrator response $y_{min}$ every time a transition from contact to slip occurs.

### 2.3. From Single-Finger to Multi-Finger Grip Force Control

When progressing towards multi-finger grip stabilization, the complexity of the tasks quickly scales accordingly with hand dexterity. Generally, the increase in degrees of freedom can be coped with either by identifying a lower-dimensional manifold for the problem or by decomposing the problem. Following the core insight in [27,28] suggesting that human multi-finger grip stabilization appears to be accomplished by separate neural circuits that interact through the object instead of via the central nervous system, we hypothesize that *multi-finger robot grip stabilization can be accomplished using the same single-finger stabilization controller on each finger independently*. In [28], three grip scenarios are compared using human subjects: (i) a grip between the index and thumb of one of the subjects hands, (ii) a grip between the two index fingers of a single subject and (iii) a grip between two index fingers belonging to two different subjects. Each of the grips was achieved with the same apparent ease, with the underlying neural control appearing to be unaffected by the specific task conditions. The latter of these three scenarios is similar to the human-robot joint stabilization that was performed in our prior work [33], with one of the human subjects replaced by a robot. As in the scenario involving the two human subjects, the object is jointly stabilized by the human and the robot without any issues.

To fully use the dexterous capabilities of a multi-fingered hand, we propose that each hand should be considered to be set of independently controlled fingers when pertaining specifically to stabilization. This independent control approach assumes that the object has been grasped in a manner in which opposition between the thumb and the remaining digits is ensured. Through this assumption, our approach obviously still requires a planning approach for grasping the object, but since no assumptions are made regarding the quality of the grasps, this planning can be reduced to a set of simple grasping policies. The same policies are used for every object, being selected only with regards to the type of grasp that is desired (two, three, four or five fingered grasp), and consist of simple movements beginning with a grasp pre-shaping, where the thumb is centered with respect to the remaining fingers. With the thumb centered, the grasp pre-shaping is concluded, and all fingers are flexed until contact with the object is achieved. Once contact between all the fingers and the object has been established, the proposed independent stabilizers are enabled, adjusting the grip on the object to ensure that it remains stable.

A set of independent fingers—in contrast to a fully connected manipulator—allows decomposing the object stabilization control problem such that each finger separately predicts future slip based on tactile sensing, counteracting it by independently adjusting the applied forces. While synchronization only through the tactile feedback may appear counter-intuitive, it actually greatly reduces the dimensionality of the control problem while ensuring that the fingers affect each other only when necessary for object stabilization. As a result, it not only becomes more straightforward to design stabilizing control laws, but the synchronization becomes more robust.

## 3. Experimental Evaluation

The proposed independent finger control law (from Section 2) is evaluated both to constructively verify the independent finger control hypothesis as well as to show that the proposed approach works sufficiently well in practice. We begin by stabilizing several objects with a varying number of fingers, using the Allegro and the Wessling hands, without any external perturbations (Section 3.4.1), and demonstrate that a control strategy working under the proposed hypothesis is able to re-stabilize objects in-hand throughout sequences of externally applied perturbations (Section 3.4.3). The presentation of the results is preceded by a detailed description of the experimental setup, i.e., robotic platform and an account of the tactile sensors mounted on the platform as well as the sensors used to measure the external perturbations (Section 3.1), and a detailed outline of the procedure used to acquire the ground truth data for the slip classifiers (Section 3.3).

### 3.1. Experimental Setup: Testing Platform and Tactile Sensors

To demonstrate our independent finger control behavior, the control scheme was implemented on two robotic hands with different dynamic and kinematic properties: The four-finger Allegro Hand and the five-finger Wessling Robotic Hand.

The Allegro Hand (Wonik Robotics, www.simlab.co.kr; Figure 1), is a lightweight four fingered hand with four joints per finger, for a total of 16 actuated degrees of freedom. The thumb has an abduction joint, two metacarpal joints (rotation and flexing) and a proximal joint. The remaining fingers do not have abduction joints and instead have a distal joint. A PD controller was used to control the robot joint positions. One end-effector was defined for each fingertip and their positions were controlled by estimating the desired joint velocities, by means of the Jacobian Pseudo-Inverse, and integrating the estimations to acquire the desired joint positions.

The Wessling Robotic Hand has five modular fingers, each with four joints where two of these four joints are coupled and cannot be moved independently (Wessling Robotics, www.wessling-robotics.de; Figure 1). A PD controller is used for joint position control and a Pseudo-Inverse Jacobian controller is used for controlling the end-effector position of each finger. The control signals are sent to a real-time machine where the conversion to torque is performed by a joint impedance controller from Wessling Robotics [37].

While the Allegro Hand has one finger fewer than the Wessling Robotic Hand, it is more compliant, and its workspace is larger than that of the Wessling Hand. The base control loops of each hand operate at different frequencies, i.e., 300 Hz and 1 kHz for the Allegro and Wessling Hand, respectively. However, despite these differences, the slip prediction-based controllers were the same, operating at a frequency of 100 Hz. Please note that each controller used slip predictors specifically trained on data from the respective fingertip sensors, the BioTac and BioTac SP respectively for the Allegro and Wessling Hands.

The BioTac and the BioTac SP tactile sensors (SynTouch Inc., www.syntouchinc.com; Figure 1) were mounted on the Allegro and Wessling Hand, respectively, and served as fingertips. Both provide multimodal responses composed of low and high frequency pressure ($P_{\mathrm{dc}}$ and $P_{\mathrm{ac}}$), local skin deformations (*E*), temperature and thermal flow ($T_{\mathrm{dc}}$ and $T_{\mathrm{ac}}$). The sensor consists of a conductive fluid captured between a pliable skin and a rigid core. The core surface is covered with impedance sensing electrodes (19 for BioTac; 24 for BioTac SP). The pressure signals are acquired by a pressure transducer, skin deformation is measured through local impedance changes measured by the electrodes and temperature is regulated by a thermistor. All data channels of the sensor are sampled at a rate of 100 Hz. The high frequency pressure signal is acquired internally by the sensor at a rate of 2.2 kHz, but is available for readout at 100 Hz, producing batches of 22 values every 10 ms. Considering all channels and the Pac batch data, the sensors output a total of 44 or 49 values every 10 ms. As previously mentioned, this difference in output prevents the same slip predictors to be used on both platforms, requiring slip predictors trained for each specific sensor.

Finally, the Optoforce OMD-D20 3D (Optoforce Ltd., www.optoforce.com) is an optical force sensor (insets of Figure 6) that was used to measure the magnitude of external perturbations applied on the objects during in-hand re-stabilization experiments. The Optoforce reconstructs the magnitude and direction of the applied force from the values of four light sensitive photodiodes that detect the amount of reflected light by interior surface diodes. The sensor has a nominal sample rate of 100 Hz.

### 3.2. Test and Training Objects

**Figure 2:**
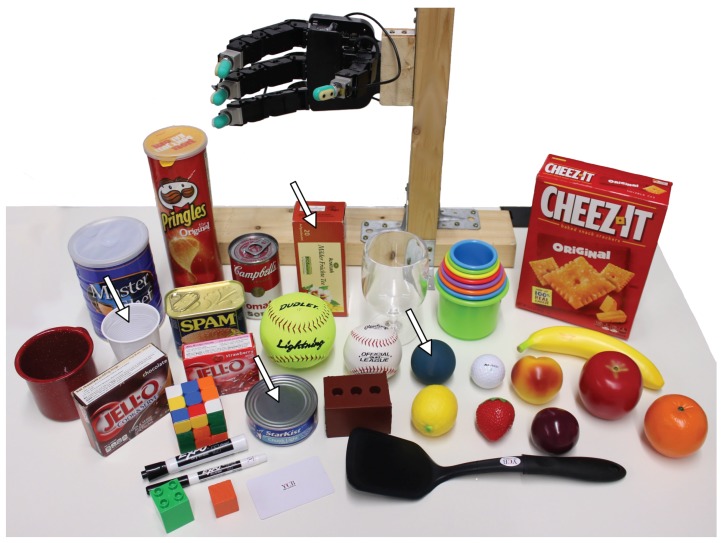
*Test objects*. Most of the objects were from the YCB object set [38] where only the tea box and the white plastic cup are not in the original set. The *training set* (indicated by the white arrows) included 4 objects only: a tuna can, a plastic cup, a ball, and a tea box.

Our set of 38 test objects belonged with two exceptions (a tea box and a plastic cup) to the YCB object set [38], and are shown in Figure 2. Among the test objects, the weight varied from 10 g to more than 400 g and grasp width from less than 10 mm to more than 100 mm. Specifically, the plastic cup was included to assess the performance of the control system when faced with highly deformable objects.

Only 4 objects were used during training: a tuna can, a plastic cup, a ball, and a tea box (arrows in Figure 2). Successful stabilization of *all* test objects thus implied that the method generalized across grasps and object properties.

### 3.3. Tactile Training

As our independent finger stabilizers reacted to slip-based feedback, it was necessary to train the classifiers responsible for slip prediction. This training required data collected on the real system and ground truth labels for the slip events.

To start data collection, one of the training objects was fixated by a support in the hand’s workspace (Figure 1). All fingers were positioned in an initial configuration and subsequently flexed until they contacted the object. Then the pressure applied by each finger was adjusted by a PID controller until a target pressure was reached on each finger. Finally, the fingers moved along the tangential contact plane, surveying the object surface. Acquiring data from three sensors simultaneously reduced the necessary number of training trials. All data from each of the fingers was concatenated into a single data set that was used to train each of the individual slip predictors. The data collection setup is exemplified in Figure 1.

**Figure 3:**
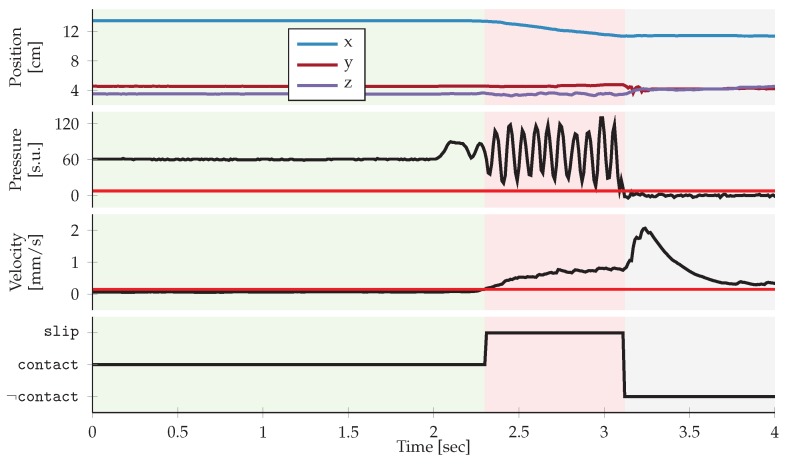
Data from the index finger during a single, representative training trial. The Cartesian instantaneous velocity was calculated from differences in finger end-effector position between two consecutive time steps. A pressure threshold, $T_{\mathrm{Contact}}$, and a movement threshold, $T_{\mathrm{Movement}}$, both indicated with red dashed lines, were used to generate the slip ground truth labels shown in the bottom panel.

Figure 3 shows a representative, single training trial with data from the index finger. Slip labels were generated automatically from the finger’s end-effector location and the recorded pressure values. The total shift in Cartesian position was calculated from the end-effector position. Since the object was fixated during training, we defined slipping as the state when the finger was in contact (i.e., the recorded pressure was above a certain threshold $T_{\mathrm{Contact}}$) and the finger was moving (i.e., the finger velocity exceeded the movement threshold $T_{\mathrm{Movement}}$; both thresholds are indicated with dashed lines in Figure 3).

This procedure relied on randomly selected velocities in task space for the object surface surveying. Target pressures were selected from 9 possible values in sensor grounded pressure units: P=[20,40,60,80,100,150,200,250,300]. Spanning the data across multiple pressures in conjunction with randomly selecting the velocity and having distinct contact locations across the three fingers allowed for training slip classifiers that were not specifically correlated with certain pressures, contact locations or fingertip velocities. In addition, all sensor values concerning pressure or finger deformation were grounded before training, preventing parametric differences in the sensors (for example nominal fluid pressure) from correlating with slip. Three trials were executed for each value of *P* on four training objects (Figure 2) for a total of 108 trials. The resulting data set thus comprised 324 single-finger trials across the three engaged fingers and was acquired in less than 15 minutes.

### 3.4. Grip Stabilization Evaluation

For the multi-finger grip stabilization scenarios, finger pressure was analyzed and used to make behavioral comparisons across objects (reported in Section 3.4.1). In addition, we assessed the in-hand re-stabilization capability of our approach as the grip was perturbed by an external agent (Section 3.4.3).

To evaluate our independence hypothesis in multi-finger grip stabilization scenarios, we begin by comparing the finger pressure profiles and used these profiles to make behavioral comparisons across objects in Section 3.4.1. We followed with an analysis of the expected stabilization success rates for a subset of the objects that exhibit different shapes, sizes and material properties in Section 3.4.2. We continued with an assessment of the in-hand re-stabilization capability of our approach as the grip was perturbed by an external agent in Section 3.4.3. We showcased how the independent finger control stabilization approach can facilitate manipulation actions via a master–slave manipulation paradigm in Section 3.4.4. Finally, we describe the current limitations of the approach in Section 3.4.5.

Since each finger was controlled independently, the approach was scalable with respect to the number of fingers. Hence, in our experiments we considered grip configurations involving two, three and four fingers when using the Allegro Hand and two, three, four and five fingers when using the Wessling Hand. The possible configurations were evenly distributed across all test objects (Figure 2) including the four objects used in the slip predictor training data collection experiments.

#### 3.4.1. Multi-Finger Grip Stabilization with Independent Finger Control

To test the validity or our independent finger control hypotheses for grip stabilization, we attempted to stabilize multiple objects with a varying number of fingers.

**Figure 4:**
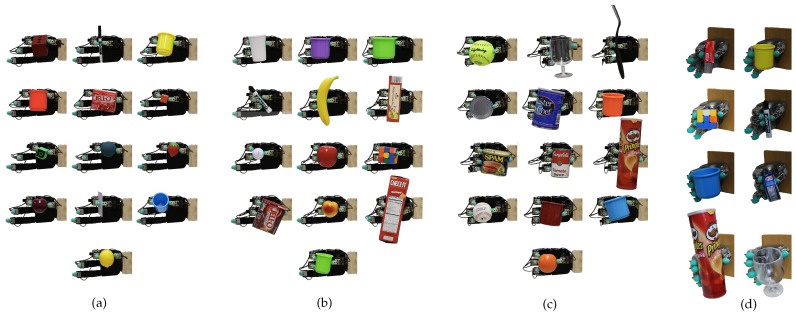
Stable grasps on a variety of objects. The specific grasp configurations varied from trial-to-trial but always resulted in stable grasps. The figure shows (**a**) two-finger, (**b**) three-finger and (**c**) four-finger grasps with the Allegro Hand and (**d**) two-finger, three-finger, four-finger and five-finger grasps with the Wessling Robotic Hand respectively from the first to the bottom lines.

We place the robotic hand in an open-hand configuration with an object positioned such that it could be held in an opposition grasp, and then closed two or more fingers (up to four with the Allegro Hand and up to five with the Wessling Robotic Hand). Immediately after all fingers have contacted the initially supported object, the grip stabilizers were activated and the independent finger stabilization process began, while the object support was removed. To ensure that the object would not be dropped during the activation transient of the grip stabilizers, each controller was initialized to generate a predefined fraction of the maximum output. For deformable objects such as the white plastic cup, this activation resulted in an initial surface deformation that was subsequently automatically reduced. Please note that for the initial grasp configuration, more advanced grasp selection approaches such as [21,22,23] could have been employed in order to increase the likelihood of the initial grasp configuration being stable. The control based on independent finger control was able to reliably and consistently stabilize all 39 test objects (Figure 4). For each object and grasp configuration (two-, three- and four-finger grasps with the Allegro Hand and up to five-finger grasps with the Wessling Hand), we recorded five trials each lasting 10 seconds with every object. A grasp was considered stable if the object was not dropped.

**Figure 5:**
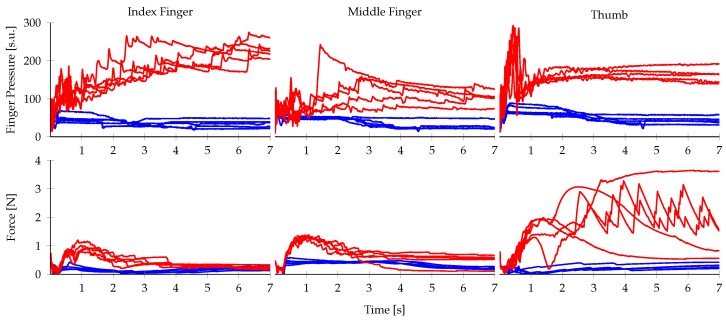
Pressure and force profiles. A comparatively light object (plastic mug; blue lines) or a heavy object (cracker box; red lines) was grasped five times with the Allegro Hand (first row) and the Wessling Robotic Hand (second row). While all attempts resulted in stable grasps, the exact configuration varied with the fingertip pressures and forces changing accordingly.

Since no desired hand configuration was enforced, the hand adopted slightly different configurations for each object and across repetitions. To study this variability in more detail, we analyzed the grip forces applied by the fingers to different objects. Figure 5 shows the pressure profiles and estimated forces for the Allegro and Wessling Hand, respectively, for trials with the lightest and one of the heaviest objects, i.e., the white plastic cup and the cracker box. The pressure profiles applied in the Allegro experiments were recorded directly from the BioTac sensors while the estimated forces applied in the Wessling experiments were calculated from joint torques and angles. The data illustrates two important emergent properties of the grasp control. First, finger pressures and forces converged to lower values when gripping the lighter plastic cup than when gripping the cracker box. Second, there was a substantial variability in force sharing between the digits across trials, particularly obvious in the profiles recorded during trials with the cracker box. Both observations can be explained straightforwardly through the design of the controller. Notably, an uncountable number of grip force distributions could result in stable grasps, but the control system did not explicitly enforce a specific distribution. Instead, pressure applied by each finger propagated through the object to the other fingers, dynamically impacting the grip force distribution while each controller minimized the risk of local slips keeping the fingertip forces low. The ability to adapt the overall grip force by reactively changing the force applied by each finger contributed to the high generalization (*Video available online:* www.youtube.com/watch?v=43uIwiFZ4I0) capability of our approach, even though no specific object orientation, weight or weight distribution was expected by the stabilizers.

#### 3.4.2. Stabilization Success Rates with Independent Finger Control

To evaluate the reliability of our proposed control scheme, we perform a quantitative analysis of the stabilization success rates on a set of objects that greatly vary in shape, size and material properties. To this end, a subset of 12 objects is selected from the full test set, and 10 stabilization trials are performed on each of the objects. The trials are performed with the Allegro Hand and its respective three possible grasp configurations. Hence, the 12 objects are split into three groups, one for each of the grasp configurations. The stabilization trials follow the procedure described in Section 3.4.1, where the stabilizers are triggered after an initial grasp and each finger attempts to find the minimum fingertip force that keeps the object firmly gripped. If the object is firmly gripped for a duration of ten seconds after the stabilizers are triggered the trial is considered a success, otherwise it is considered a failure. The observed stabilization success rates are reported in Table 1.

For objects with planar opposing surfaces such as the card, the orange cube, the JELL-O Choc box and the Cheez-It box, we observed average stabilization rates consistently close to 100%, despite the large differences in size and weight between the objects. On the other hand, while cylindrical objects such as the red cup, the white plastic cup and the Pringles box display success rates in a similar range as the planar objects, the stabilization process is harder, as the contact surfaces are smaller and the elongated nature of the cylindrical objects causes rotational slips, which are not compensated by our controllers. The effects of rotational slips become even more evident for spherical objects such as the plum and the apple and for irregular objects such as the banana, the glass and the spatula. While acquiring large contact surfaces is possible with the glass and the apple due to their sizes, only small contact surfaces are achievable with the remaining spherical and irregular objects. These small contact surfaces and the aforementioned rotational slip effects translate into lower stabilization success rates. The limitations of the approach will be discussed in more detail in Section 3.4.5.

#### 3.4.3. Grip Stabilization under External Perturbations

**Figure 6:**
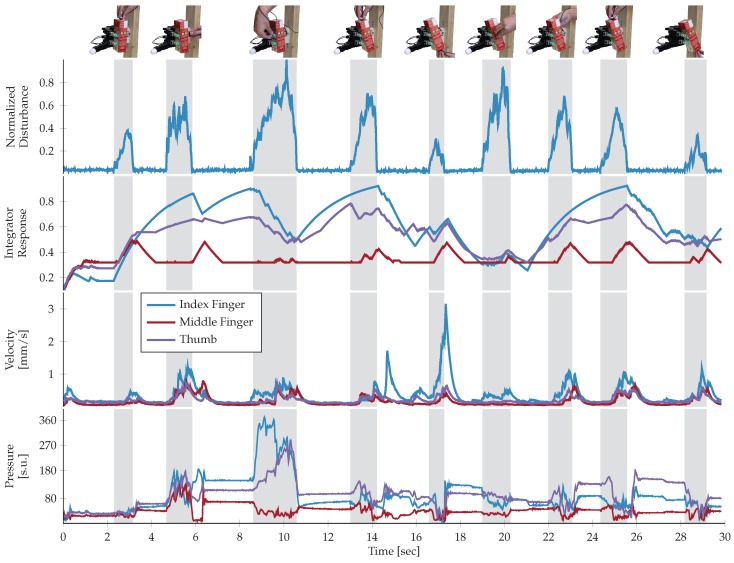
The magnitude of the external perturbations (top panel), the resulting integrator responses (second panel), changes in fingertip positions (third panel), and the fingertip pressures (bottom panel) during a full 30 second experimental run. Early pressure changes represent the initial stabilization of the object in-hand. Subsequently, the individual fingertip pressures dynamically and independently change and the integrator response increases every time that the object was perturbed by the experimenter (top panel), or, interestingly, by ’spontaneous’ changes in individual fingers (e.g., around 17 seconds).

To further test the validity of our control hypothesis, we investigated responses to externally applied perturbations (Figure 6). Once the object was stabilized in the robotic hand, the experimenter held an Optoforce sensor and used it to disrupt the object state by applying sequences of irregular disturbances (*Video available online:* www.youtube.com/watch?v=0wj3RWXyOCk), either to the different surfaces of the objects or to the fingertips, during 30 second recording periods (insets in Figure 6).

For the entire duration of these experiments, the stabilizers invariably counteracted the perturbations successfully by adapting the finger pressures. With every perturbation, we observed a change in the fingertip forces and an increase in the accumulated value of the integrator that regulated the applied velocity. As a result, the individual fingers applied slightly different steady-state forces after each perturbation. For instance, the 1st, 4th and 8th perturbation in Figure 6 were applied in a similar fashion (i.e., from top) but in response, the independent finger controllers generated different stable grip force distributions. Indeed, while the object was held in a similar position throughout this trial, the pressure distributions across the fingers differed following each perturbation. Changes in fingertip forces due to slip prediction noise or re-stabilization were also frequently observed (e.g., around 16 and 21 second mark). Nonetheless, the ability to predict slip, as opposed to detecting it after its initial occurrence, allows each finger to start counteracting the perturbations as soon as they are applied to the object. Coupled with the smoothness introduced by the integrator, the controllers avoid large and instantaneous changes in force that could potentially render the multi-finger system unstable, but are still able to prevent the loss of contact due to slip events.

#### 3.4.4. Master–Slave Operation

**Figure 7:**
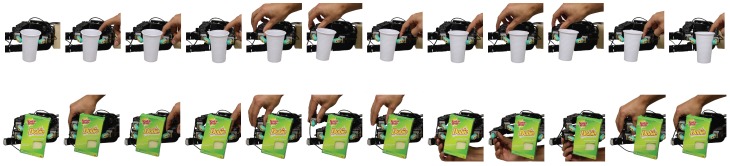
Experiments showcasing master–slave operation where the fingers stabilize the object despite one finger introducing perturbations to change the object’s positions in-hand. The experiment showcases how the independent finger grip stabilization controllers, paired with upper level control policies, can enable in-hand object manipulation. Instead of an upper level controller, finger perturbations were introduced by a human experimenter for a two-finger grasp (upper row) and a three-finger grasp (lower row). In addition, in the three-finger grasp we show that fingers can be removed from the object while it is re-stabilized by the remaining fingers.

From the perspective of the independent fingertip controllers, there was no conceptual difference between external perturbations and those caused by the actions of other fingertips. This interaction was further explored in a manipulation experiment, where using a master–slave manipulation paradigm, the object is manipulated by having several fingers stabilize the object while other fingers disturb the object position. To achieve this, an experimenter manually pushed or pulled a finger to increase or decrease the force it applied, while the controllers of the remaining fingers jointly stabilized the grasp. Indeed, two-, three- and four-digit grasps remained stable while the object position changed (*Video available online:* www.youtube.com/watch?v=sEI3uud9wgw). In addition, for grasps with more than two digits, even when one of the digits was lifted off the surface of a grasped object, the remaining stabilizers kept the object stable by redistributing the force among the fingers that remained in contact. In contrast to more traditional solutions for manipulation control, force sharing between the engaged fingers varied substantially from trial-to-trial due to the emerging nature of the independent finger control policy. Such variability is, however, typical in human manipulation [27,28,29,39]. While it could be easily removed by additional regularization, it could actually be beneficial in practice as it allows a wider range of potential solutions (e.g., for use in a manipulation planner). The disturbances applied by the human experimenter in the master–slave manipulation experiments are shown in Figure 7.

**Figure 8:**
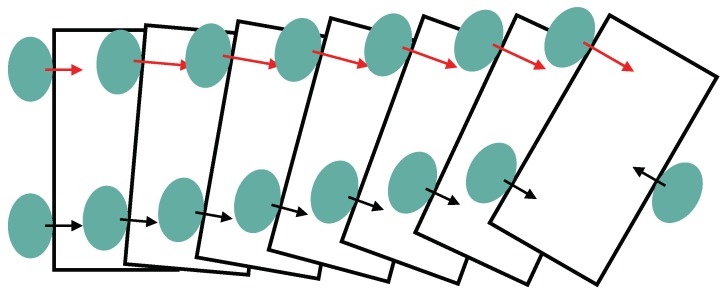
An example of how a rotation can be achieved with a tripod grasp when relying on a master–slave control approach. In this case, the index finger is the master, increasing the amount force applied in its normal contact direction. This force increase forces the object to pivot around the center of the grasp while the remaining fingers keep the object stable throughout the movement.

The results of this master–slave manipulation experiment suggest that an independent fingertip control scheme could potentially be used as the base level of a hierarchical control framework. Performing above such a lower-level scheme, could enable higher-level control policies to perform complex manipulations by applying a set of perturbations that would move the object to the target configurations while the independent stabilizers ensure that the object remains firmly gripped. In a basic scenario, rotating an object that is held in a tripod grip between the index and middle fingers and the thumb, would simply require an increase in the force applied by either the index or middle fingers, depending on the desired rotation, while the remaining fingers simply rotate with the object, keeping it stable. An illustrative example of a rotation where the index finger is the master is depicted in Figure 8. In this example, an increase in normal force applied by the index finger, here considered the master finger, causes the object to pivot along a tripod grasps center axis. Since the remaining fingers only wish to prevent slip, they act as the slaves, keeping the object stably gripped throughout the movement.

#### 3.4.5. Current Limitations

In the previous sections we showcased several properties of our approach that are desirable for in-hand grip stabilization and in-hand manipulation. Despite these properties, our approach still fails in specific scenarios.

One of such scenarios is when stabilizing objects that due to their shape or weight distributions, are susceptible to gravity induced torques that cause rotational slips between the object and the fingertips. Compensating for rotational slips not only requires larger normal forces, due to much smaller rotational coefficients of friction, but currently our slip predictors are also not able to detect or predict rotational slip. While collecting training data for rotational slip, and using it to training our predictors could potentially alleviate this problem, rotational and translational slips are physically correlated [40], and it is unclear if the current slip prediction approach is able to cope with these correlations. The inability to compensate for rotational slips is particularly relevant when the contact surface between the fingers and the object is small, with any form of slip causing the object to drop immediately.

One other fail case is related to the current estimation of the contact normal, $N_{t}$. The current normal estimation was proposed in [36], and while it is stable for quasi-static point contacts, it quickly becomes unstable whenever contact shifts and the deformation of the fingertip surface completely changes. This is particularly detrimental for the stabilization of irregular objects such as the banana, where the irregular surfaces of the objects translate to large changes in finger deformation even for small control actions. This is directly observable from the success rates reported for this object in Table 1.

## 4. Conclusion and Discussion

The proposed independent finger grip stabilization control approach, inspired by findings of human neurophysiological research, was able to stabilize a wide range of objects by taking advantage of the generalization capabilities of the slip feedback signals and of the modular nature of the control scheme. The approach not only produced grips that kept the objects stable within the hand and were robust to perturbations but also displayed fairly high stabilization success rates across objects of different sizes, shapes and material properties. Results from a manipulation experiment using a master–slave paradigm also suggest that such a control scheme, when used as the lower-level of an hierarchical control approach, could facilitate the design of higher-level control policies that are able to manipulate objects in-hand.

### 4.1. Summary of the Contribution

We have corroborated the hypothesis that stable grips can emerge while using a control scheme where a set of independent finger controllers is distributed among the available fingers. Indeed, the synchronization between fingers emerge from the tactile feedback of each finger controller and enable stable gripping despite disturbances caused by poor contact distribution on the fingertip surfaces, introduced by other fingers action on the object, or external disturbances. Each finger thus automatically compensated for changes that jeopardized grasp stability. Moreover, our modular control approach was shown to be generalizable across multiple objects, even objects that were substantially different from the objects in the training set.

### 4.2. Recognized Shortcomings

Using the low-dimensional slip signals defined in previous work [33], enabled the design of the controller used in this paper. As the full tactile state is much richer than the slip signals, we may potentially have discarded relevant information.

Additionally, in the proposed approach we focused on ’low-level’ control of grasp stability. As such, the objects tested were provided to the hand in configurations where the stabilization would be possible, requiring neither finger gating nor re-positioning. Despite not directly addressing finger gating and finger re-positioning to transition from configurations where stabilization is not possible, both were shown to be easily achievable from stable grip configurations in the master–slave manipulation experiments.

The implemented controller is reactive, albeit that upcoming slips are *predicted* by the controller. The temporal limitations in this respect have not been analyzed. For comparison, it takes human as much as 60–80 ms to initiate force responses to incipient and overt fingertip slips and at least 50–100 ms to generate substantial counteracting forces [41,42], i.e., these delays are too long for preventing the loss of a stable grasp once overt slippage occurs.

### 4.3. Future Work

Partitioning the hand into a set of independent fingers allows the manipulation problem to be viewed as a distributed problem where each finger solves the task locally and coordination only emerges by interaction through the object. This setting invites simpler control models than when considering a complete model for the full hand. Specifically, we consider it realistic to use data-driven approaches that take into account a richer sensor space, as the dimensionality of the problem is distributed across the fingers. Our future work will focus on exploring the high dimensionality of the feedback signals and learning stabilization controllers using reinforcement learning approaches in these high-dimensional spaces. Learning such stabilization controllers could potentially address the fail cases reported in Section 3.4.5, by directly learning how to estimate the direction of the stabilization action and also how to compensate for rotational slips.

Finally, for complex manipulations, we propose that independently controlling the fingers will be necessary but not sufficient to achieve robust performance. Using the independent control as the base level in a hierarchical control framework is expected to enable higher-level control policies to perform these manipulations, effectively creating a robust control hierarchy, where the task complexity is distributed across the several levels of the hierarchy. Building such a hierarchy is thus a potentially interesting future work.

## Figures and Tables

**Table 1:** Assessment of the stabilization success rates for several objects. Ten stabilization trials are performed for each object and the rate of successful stabilization is reported.

**Two-Finger Grasp**			
Card	Orange Cube	Plum	Red Cup
90%	100%	80%	70%
**Three-Finger Grasp**			
White Plastic Cup	JELL-O Choc	Banana	Apple
90%	100%	50%	60%
**Four-Finger Grasp**			
Glass	Pringles	Spatula	Cheez-It
90%	80%	60%	100%
